# Sex-Specific Salivary Biochemical Composition in a Healthy Population

**DOI:** 10.3390/ijms27052214

**Published:** 2026-02-26

**Authors:** Elena A. Sarf, Kirill S. Yunkind, Denis V. Solomatin, Lyudmila V. Bel’skaya

**Affiliations:** 1 Biochemistry Research Laboratory, Omsk State Pedagogical University, 644099 Omsk, Russia; sarf_ea@omgpu.ru (E.A.S.); safety@omgpu.ru (K.S.Y.); 2 Department of Mathematics and Mathematics Teaching Methods, Omsk State Pedagogical University, 644099 Omsk, Russia; solomatin_dv@omgpu.ru

**Keywords:** saliva, healthy control, sex, biochemistry, amino acids, lipids, cytokines, electrolytes

## Abstract

The high potential of saliva for use in the non-invasive diagnosis of a number of diseases raises a number of questions regarding the substantiation of normal and abnormal salivary composition criteria. Factors that must be considered when forming patient cohorts include age, hormonal status, and circadian variability. However, the influence of sex remains controversial. The aim of this study was to investigate the influence of sex on the biochemical composition of normal saliva, including amino acid and lipid profiles, cytokine levels, and electrolytes. The study involved 120 healthy volunteers (75 females and 45 males). The amounts of electrolytes (NH_4_^+^, K^+^, Na^+^, Mg^2+^, Ca^2+^, Cl^−^, SO_4_^2−^, NO_2_^−^, NO_3_^−^, F^−^, PO_4_^3−^), amino acids (Arg, Lys, Tyr, Phe, His, Leu+Ile, Met, Val, Pro, Thr, Ser, Ala, Gly), cytokines (VEGF, MCP-1, TNF-α, IL-1β, IL-2, IL-4, IL-6, IL-8, IL-10, IL-18, INF-α, INF-γ), and biochemical parameters (protein, urea, total content of α-amino acids, imidazole compounds, lipid peroxides) were analyzed. Lipid content was determined based on the intensity of absorption bands at 1396, 1458, 2853, 2923, and 2957 cm^−1^ in the IR spectra of salivary lipid extracts. A clear sex correlation was found for amino acid and lipid content in saliva. For electrolytes and biochemical parameters, median differences were demonstrated in some cases; however, the range of variation for all parameters overlapped. Although overall cytokine profiles did not show clear multivariate separation, significant differences between sexes were observed for individual cytokines (IL-1β and IL-10). A comprehensive assessment of all parameters (amino acids, lipids, cytokines, etc.) allows for the formation of a sex-associated metabolic profile of saliva. Therefore, it is recommended to avoid the use of mixed cohorts when analyzing the amino acid and lipid profiles of saliva.

## 1. Introduction

Saliva as a biological fluid has significant potential in the non-invasive diagnosis of a number of systemic diseases, cardiovascular diseases, systemic and local inflammation, endocrinological and metabolic disorders [1,2,3,4]. Mixed saliva contains electrolytes, proteins, lipids and enzymes, bacteria, and epithelial cells [5,6]. Proteins and other substances enter saliva via the bloodstream, and in some cases there is a close relationship between their concentrations in saliva and serum [7]. The salivary glands are highly vascularized, and an exchange of compounds occurs, passing by passive diffusion or active transport from blood to saliva and vice versa [8]. Salivary lipids are mainly secreted by the major salivary glands, but some lipids, such as cholesterol and some fatty acids, diffuse directly from serum into saliva [9]. The composition of saliva and its functional role in oral hygiene is of interest in the context of dietary and physicochemical aspects of oral health [10]. The use of salivary biomarkers represents an interesting opportunity for the implementation of diagnostic and patient monitoring strategies, limiting the risks associated with more invasive procedures [11,12].

The use of saliva in clinical laboratory diagnostics is limited by the lack of standardization of procedures for collection, storage, transportation, and the preanalytical stage [13]. It is known that the composition of saliva can be influenced by various factors: circadian rhythm [14,15], which determines the volume of saliva and its quantitative and qualitative composition, age [16,17], sex [18], diet, oral hygiene, hormonal changes, and many others [19,20]. Literary data on the influence of sex on the biochemical parameters of saliva are particularly contradictory; a number of indicators can change (IL-6, TNF-α, MUC5B, secretory IgA, and chitinase activity) [7], while for others, no relationship with sex was found (IL-1β, CRP, albumin levels, cystatin S, α-amylase) [21]. Thus, information on the influence of sex on the biochemical composition of saliva is scarce and scattered. There is no published summary information on analytes of different classes measured simultaneously in saliva when taking into account sex.

The aim of this study was to examine the influence of sex on the biochemical composition of saliva in normal individuals, including the amino acid and lipid profile and the content of cytokines, electrolytes, etc.

## 2. Results

As the first stage, the salivation rate was determined. The salivation rate was 0.88 [0.85; 0.91] mL/min for males and 0.83 [0.79; 0.92] mL/min for females (*p* > 0.05). Due to the lack of statistically significant differences in salivation rate, this indicator was not used in further calculations.

### 2.1. The Content of Cations and Anions in Saliva Depending on Sex

**Figure 1:**
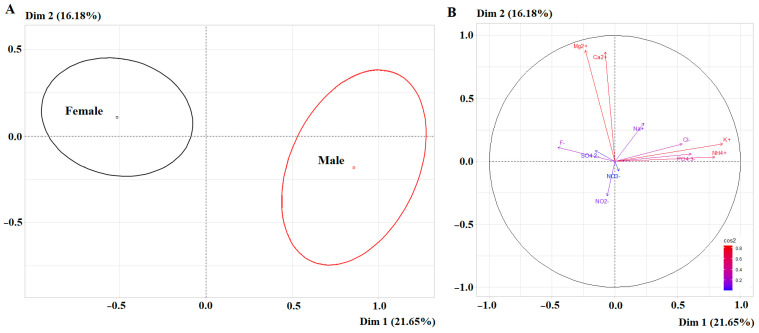
PCA factorial diagram (**A**) and correlation circle (**B**) comparing cation and anion content in saliva depending on sex (*p* = 2.908 × 10^−6^).

Statistically significant differences between the subgroups of different sexes were shown by the PCA method (*p* < 0.0001) (Figure 1A). High correlation coefficients for the first principal component were shown for potassium (*r* = 0.8592) and ammonium (*r* = 0.7965) ions, while for the second principal component they were shown for magnesium (*r* = 0.8838) and calcium (*r* = 0.8725) ions (Figure 1B).

An individual comparison revealed that males had higher levels of ammonium, sodium, and potassium ions in their saliva, while levels of magnesium, sulfate, and fluoride ions were lower (Table 1). Additional calculation of the Ca/P and Na/K ratios revealed that, despite slightly higher values for both parameters in the female subgroup, there were no statistically significant differences between the subgroups (Table 1).

**Figure 2:**
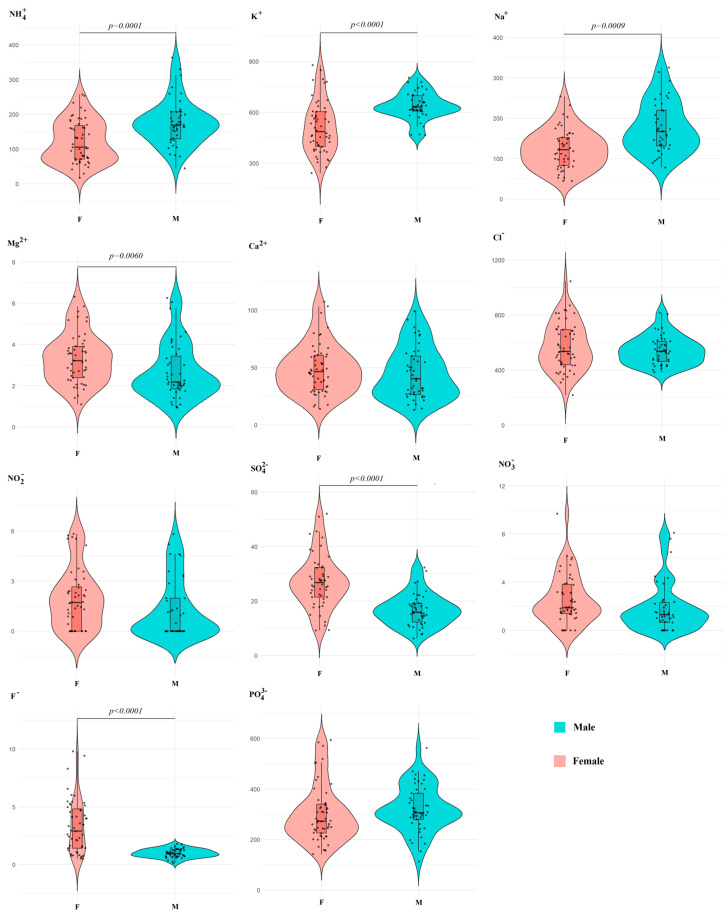
Violin plots for pairwise comparisons of cation and anion concentrations in saliva depending on sex. Black dots are individual values, black boxes are interquartile range, the line inside the box is the median.

Figure 2 shows that statistically significant differences in medians were present, but in general the range of values (minimum–maximum) for most electrolyte indicators of saliva overlapped for volunteers of different sexes, with the exception of potassium, sulfate and fluoride ions (Figure 2).

### 2.2. Salivary Amino Acid Profile Depending on Sex

It was found that the concentration of amino acids in female saliva was statistically significantly higher than that of males for all amino acids except Val (Table 2). Despite pronounced sex-related differences, the calculation of amino acid ratios revealed that sex differences were less pronounced for these amino acids. For example, the Arg/Lys ratio was 0.38 ± 0.18 and 0.34 ± 0.11 for males and females, respectively; no statistically significant differences were found between sex subgroups.

**Table 2:** Content of amino acids in saliva.

Amino Acids	Female, *n* = 71		Male, *n* = 45		*p*-Value
	Me [IQR]	Min–Max	Me [IQR]	Min–Max	
Arg, nmol/L	12.55 [5.00; 28.50]	0.00–130.65	5.98 [2.95; 15.64]	0.00–35.07	0.0013 *
Lys, nmol/L	55.21 [27.52; 93.71]	3.69–457.01	24.76 [16.54; 31.72]	3.72–70.87	<0.0001 *
Tyr, nmol/L	36.50 [18.60; 53.78]	0.00–488.27	16.99 [10.34; 23.75]	2.93–54.21	<0.0001 *
Phe, nmol/L	26.01 [14.67; 38.69]	0.00–122.22	10.72 [7.02; 14.99]	3.88–29.38	<0.0001 *
His, nmol/L	24.77 [13.86; 52.10]	0.00–262.05	15.89 [9.76; 28.02]	3.30–103.18	0.0079 *
Leu+Ile, nmol/L	36.92 [18.52; 62.95]	0.00–201.10	17.88 [11.39; 22.97]	5.72–40.80	<0.0001 *
Met, nmol/L	91.62 [47.77; 173.18]	6.32–794.85	36.26 [23.32; 65.37]	3.06–185.98	<0.0001 *
Val, nmol/L	18.69 [5.56; 35.45]	0.35–341.67	19.93 [2.69; 34.16]	0.48–71.30	0.9030
Pro, nmol/L	118.04 [44.91; 271.52]	5.45–1880.48	30.85 [20.36; 49.82]	9.82–196.99	<0.0001 *
Thr, nmol/L	13.56 [6.62; 37.26]	0.30–141.20	5.22 [3.92; 8.38]	0.00–233.29	<0.0001 *
Ser, nmol/L	32.35 [16.24; 82.06]	1.11–418.31	16.09 [11.61; 21.89]	5.32–73.47	0.0002 *
Ala, nmol/L	70.84 [32.94; 133.24]	2.13–407.45	39.94 [23.82; 63.18]	10.87–184.08	0.0017 *
Gly, nmol/L	238.58 [116.96; 444.52]	22.23–2569.60	124.04 [84.03; 179.70]	20.31–366.59	0.0003 *

*—differences between subgroups are statistically significant, *p* < 0.05.

**Figure 3:**
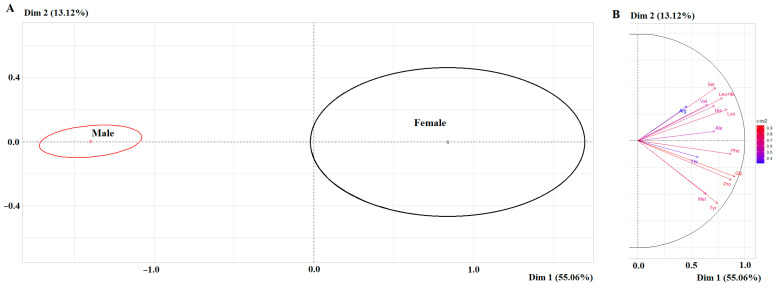
PCA factorial diagram (**A**) and correlation circle (**B**) comparing amino acid content in saliva depending on sex (*p* = 2.802 × 10^−5^).

Differences between the subgroups of males and females in the amino acid profile are also statistically significant (Figure 3A). Moreover, significant correlation coefficients are shown only for the first principal component for Gly (*r* = 0.9089), Pro (*r* = 0.8744), Phe (*r* = 0.8738), Lys (*r* = 0.8353), Leu+Ile (*r* = 0.7919), Tyr (*r* = 0.7434), Ser (*r* = 0.7281), Ala (*r* = 0.7201) and His (*r* = 0.7164) (Figure 3B).

**Figure 4:**
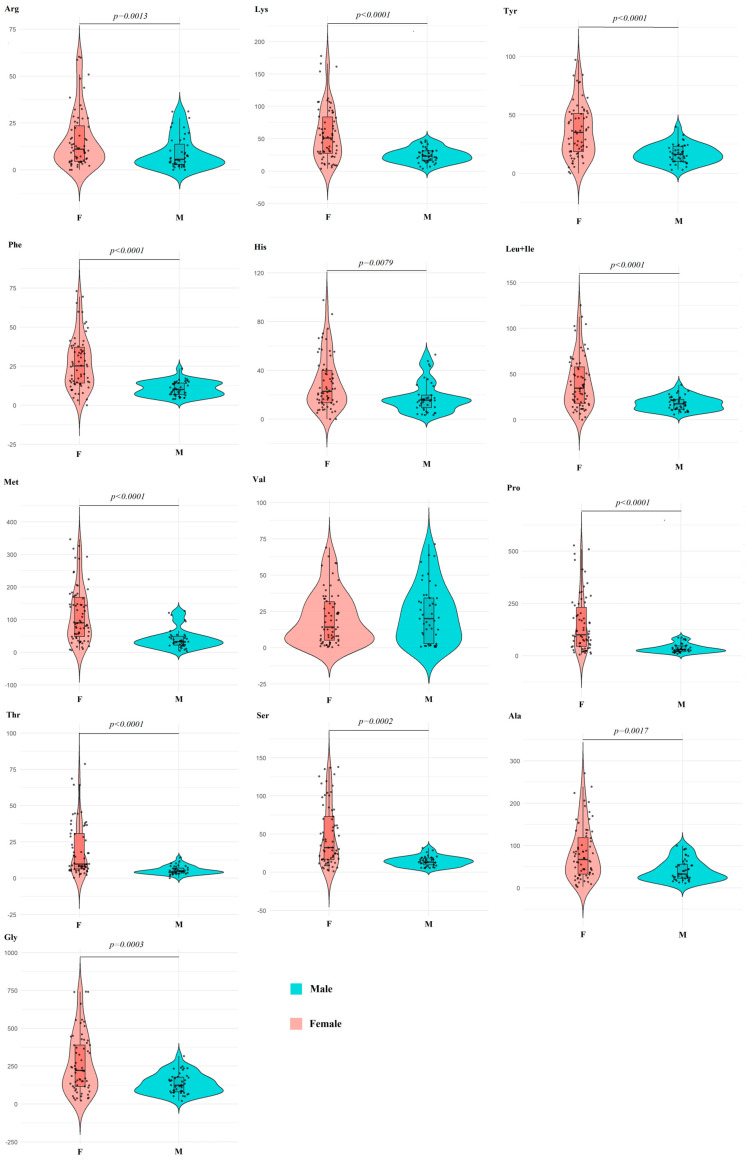
Violin plots for pairwise comparison of amino acid concentrations in saliva depending on sex. Black dots are individual values, black boxes are interquartile range, the line inside the box is the median.

It should be noted that the range of values for the content of individual amino acids in the female group is much wider than in the male group, with the exception of Val (Figure 4).

### 2.3. Lipid Profile of Saliva Depending on Sex

**Figure 5:**
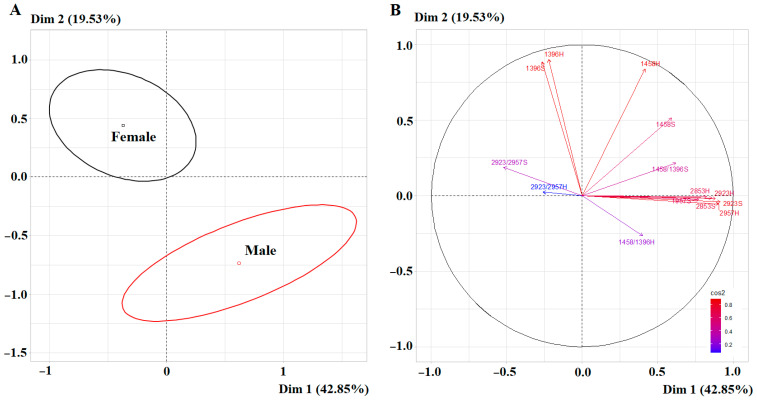
PCA factorial diagram (**A**) and correlation circle (**B**) comparing lipid content in saliva depending on sex (*p* = 4.743 × 10^−5^).

The intensity of the absorption bands at 1396 and 1458 cm^−1^ in the female subgroup was statistically significantly higher than in the male subgroup (+51.9%, *p* = 0.0034 and +32.9%, *p* = 0.0024, respectively) (Table 3). On the other hand, for the absorption bands at 2853, 2923 and 2957 cm^−1^, the opposite trend was observed (−19.8%, −15.2% and −20.0%, *p* = 0.0053, respectively) (Table 3). The separation of the subgroups by sex was statistically significant based on the combination of salivary lipid absorption bands (Figure 5A). In this case, the correlation circle of the PCA diagram showed high correlation coefficients for the first principal component for 2923S cm^−1^ (*r* = 0.9156), 2957H cm^−1^ (*r* = 0.9049), 2923H cm^−1^ (*r* = 0.8852), 2853S cm^−1^ (*r* = 0.8622), 2853H cm^−1^ (*r* = 0.8260) and 2957S cm^−1^ (*r* = 0.7737) (Figure 5B). For the second principal component, high correlation coefficients were shown for 1396H cm^−1^ (*r* = 0.9041), 1396S cm^−1^ (*r* = 0.8876) and 1458H cm^−1^ (*r* = 0.8396) (Figure 5B).

**Table 3:** Characteristics of lipid absorption bands in the IR spectra of saliva.

AB, cm^−1^		Female, *n* = 59		Male, *n* = 45		*p*-Value
		Me [IQR]	Min–Max	Me [IQR]	Min–Max	
1396	H	0.79 [0.42; 1.59]	0.23–2.66	0.52 [0.44; 0.66]	0.07–1.46	0.0034 *
	S	4.63 [2.61; 10.00]	0.99–19.20	2.84 [2.33; 3.47]	1.06–7.73	0.0001 *
1458	H	2.75 [1.91; 4.04]	1.12–5.22	2.07 [1.77; 2.25]	1.01–12.40	0.0024 *
	S	12.00 [7.60; 18.90]	4.56–72.80	8.36 [7.53; 9.40]	4.15–252.00	0.0078 *
2853	H	6.89 [6.12; 9.63]	2.21–18.70	8.59 [6.65; 12.00]	3.78–20.20	0.0502
	S	127.0 [110.0; 195.0]	39.6–402.0	172.0 [122.0; 247.0]	67.8–477.0	0.0352
2923	H	11.70 [10.30; 16.60]	1.66–32.60	13.80 [11.00; 19.90]	1.40–34.70	0.1247
	S	299.0 [263.0; 455.0]	16.7–846.0	370.0 [285.0; 508.0]	164.0–951.0	0.0707
2957	H	2.48 [2.09; 3.98]	0.62–12.10	3.10 [2.82; 3.81]	1.89–8.53	0.0053 *
	S	20.10 [15.90; 66.80]	5.1–259.0	45.30 [35.20; 65.10]	13.8–393.0	0.0004 *
2923/2957	H	4.46 [3.74; 5.20]	2.29–7.44	4.13 [3.69; 5.03]	0.47–6.63	0.3267
	S	13.83 [6.56; 16.53]	2.67–33.48	7.80 [5.46; 11.31]	0.71–19.19	0.0049 *
1458/1396	H	3.00 [2.21; 4.79]	1.72–10.44	4.07 [3.52; 4.46]	2.35–26.14	0.0111 *
	S	2.09 [1.75; 3.25]	1.15–21.54	2.96 [2.63; 3.53]	2.12–54.31	0.0004 *

Note. AB—absorption band; H—intensity (height); S—area. *—differences between subgroups are statistically significant, *p* < 0.05.

**Figure 6:**
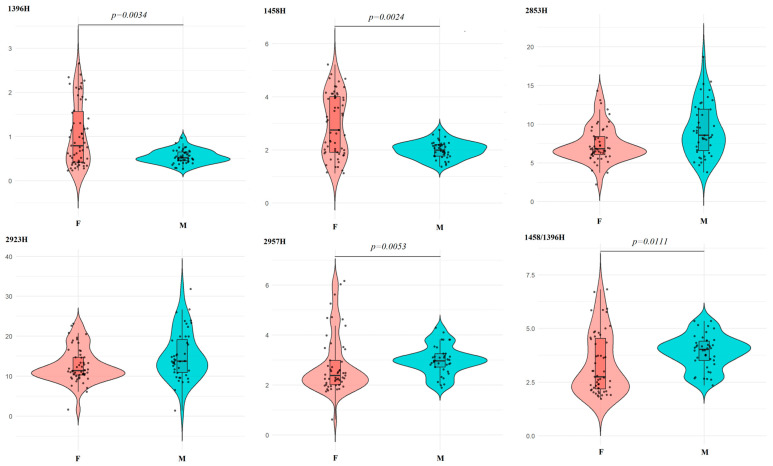
Violin plots for pairwise comparison of salivary lipid concentrations by sex. Black dots are individual values, black boxes are interquartile range, the line inside the box is the median.

As with amino acids, a narrower distribution of IR spectral characteristics associated with the lipid profile of saliva was shown in male volunteers (Figure 6). It should be noted that for the intensity ratio of 2923/2957 cm^−1^, which reflects the ratio of unbranched and branched lipid and fatty acid molecules (CH_2_/CH_3_), only the difference in absorption band areas, but not the intensities, was statistically significant (Table 3). However, for the ratio of 1458/1396 cm^−1^, the differences were significant in both cases, but the variation ranges completely overlapped (Figure 6).

### 2.4. Cytokine Levels in Saliva Depending on Sex

**Figure 7:**
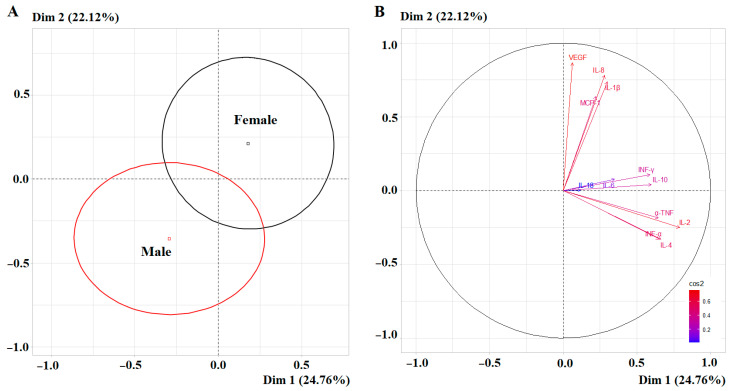
PCA factorial diagram (**A**) and correlation circle (**B**) comparing cytokines content in saliva depending on sex (*p* = 0.0727).

Unlike the other parameters, PCA did not reveal statistically significant differences between the subgroups of different sexes in cytokine concentrations: complete separation was not observed for either the first or second principal components (Figure 7A). However, high correlation coefficients for the first principal component were shown for IL-2 (*r* = 0.7901), and for the second principal component for VEGF (*r* = 0.8675), IL-8 (*r* = 0.7835), and IL-1β (*r* = 0.7391) (Figure 7B).

**Figure 8:**
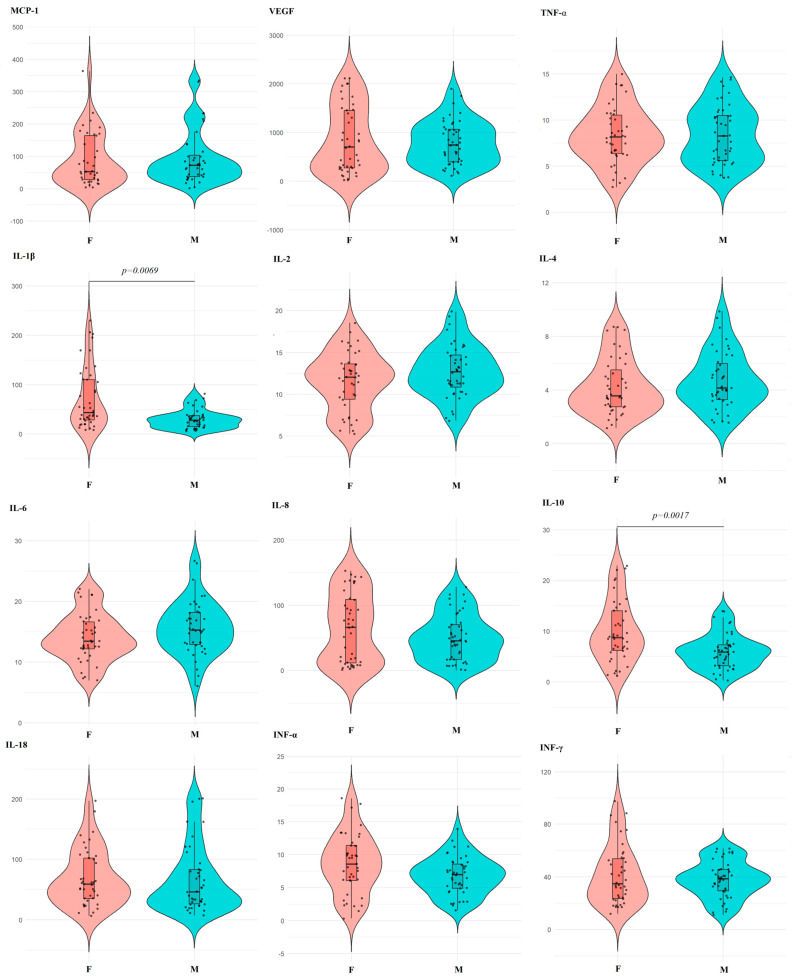
Violin plots for pairwise comparison of salivary cytokine concentrations by sex. Black dots are individual values, black boxes are interquartile range, the line inside the box is the median.

Only two cytokines showed higher levels in saliva in women: IL-1β (+48.2%, *p* = 0.0069) and IL-10 (+47.6%, *p* = 0.0017) (Table 4). For the remaining cytokines, individual differences by sex were not pronounced, and the variation ranges overlapped (Figure 8).

**Table 4:** Cytokine levels in saliva depending on sex.

Cytokines	Female, *n* = 44		Male, *n* = 45		*p*-Value
	Me [IQR]	Min–Max	Me [IQR]	Min–Max	
MCP-1, pg/mL	78.46 [30.38; 193.1]	4.23–1340.4	77.31 [41.15; 175.8]	1.92–667.31	0.9607
VEGF, mU/mL	700.8 [271.9; 1460.4]	20.8–2115.4	744.6 [396.9; 1068.5]	111.5–1898.5	0.8024
α-TNF, pg/mL	8.24 [6.41; 10.73]	2.71–20.50	8.28 [5.61; 10.50]	3.70–14.62	0.7396
IL-1β, pg/mL	45.27 [28.52; 115.0]	8.20–239.69	30.55 [15.55; 46.41]	5.78–182.66	0.0069 *
IL-2, pg/mL	12.17 [9.47; 14.41]	5.26–28.29	12.76 [11.05; 15.00]	6.84–21.18	0.2934
IL-4, pg/mL	3.62 [2.76; 5.87]	1.16–17.41	4.15 [3.37; 6.60]	1.56–22.28	0.2277
IL-6, pg/mL	13.48 [12.19; 17.02]	6.97–48.20	15.39 [13.03; 19.10]	6.07–74.49	0.0965
IL-8, pg/mL	66.26 [11.08; 109.3]	1.16–152.77	45.36 [16.91; 70.33]	0.59–127.90	0.3432
IL-10, pg/mL	8.96 [6.29; 15.07]	1.18–33.84	6.07 [3.18; 7.62]	0.29–14.73	0.0017 *
IL-18, pg/mL	61.27 [35.44; 109.7]	6.58–811.79	52.83 [29.29; 111.8]	7.21–324.50	0.5141
INF-α, pg/mL	9.46 [6.16; 12.95]	0.36–73.57	7.14 [5.00; 8.93]	1.61–55.18	0.0503
INF-γ, pg/mL	36.52 [23.74; 56.15]	11.89–142.63	38.56 [29.30; 45.59]	11.15–61.52	0.6936

*—differences between subgroups are statistically significant, *p* < 0.05.

### 2.5. Biochemical Parameters of Saliva Depending on Sex

**Figure 9:**
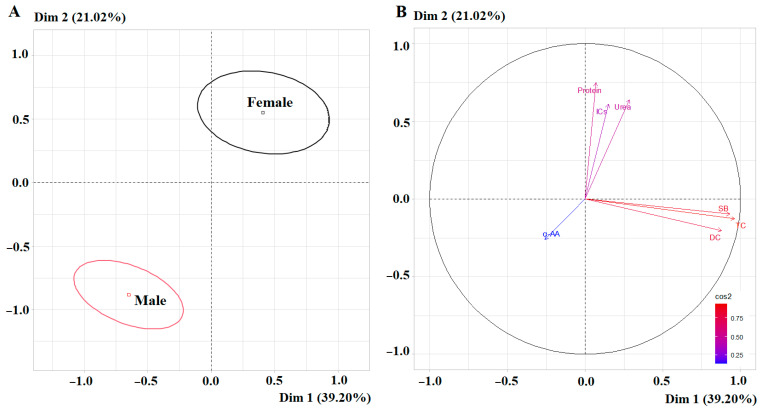
PCA factorial diagram (**A**) and correlation circle (**B**) comparing biochemical indicators in saliva depending on sex (*p* = 1.315 × 10^−14^).

The first principal component showed high correlation coefficients with the level of DC (*r* = 0.9625), TC (*r* = 0.9328) and SB (*r* = 0.8780), and the second principal component showed high correlation coefficients with the concentration of total protein (*r* = 0.7467) (Figure 9B).

**Figure 10:**
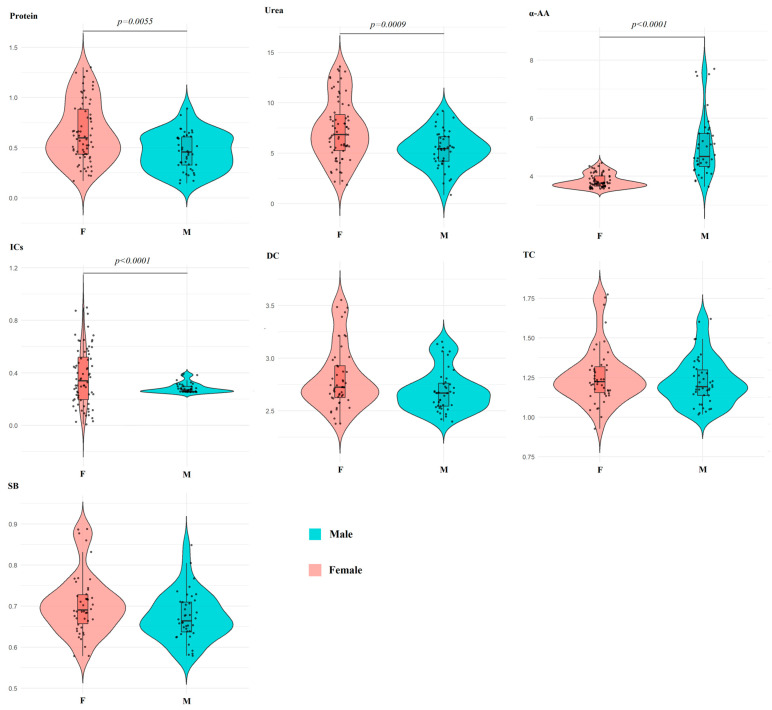
Violin plots for pairwise comparison of biochemical indicator concentrations in saliva depending on sex. Black dots are individual values, black boxes are interquartile range, the line inside the box is the median.

Pairwise comparison revealed no statistically significant differences in the levels of lipid peroxidation products, but differences in protein, urea, total α-amino acid, and imidazole levels were statistically significant (Table 5). The range of values around the median for the female subgroup was slightly wider than for the male subgroup; however, in both cases, they were within normal limits (Figure 10).

**Table 5:** Biochemical composition of saliva depending on sex.

Indicators	Female, *n* = 75		Male, *n* = 45		*p*-Value
	Me [IQR]	Min–Max	Me [IQR]	Min–Max	
Protein, g/L	0.62 [0.44; 0.91]	0.17–2.09	0.47 [0.33; 0.62]	0.15–1.20	0.0055 *
Urea, mmol/L	6.85 [5.25; 9.39]	1.87–16.43	5.47 [4.21; 6.66]	0.86–10.49	0.0009 *
α-AA, mmol/L	3.76 [3.65; 4.02]	3.56–5.16	5.02 [4.39; 5.89]	3.63–12.63	<0.0001 *
ICs, mmol/L	0.339 [0.195; 0.526]	0.008–0.898	0.250 [0.040; 0.641]	0.002–0.854	<0.0001 *
DC, c.u.	2.73 [2.63; 3.01]	2.38–4.03	2.67 [2.55; 2.81]	2.40–3.36	0.0600
TC, c.u.	1.24 [1.17; 1.42]	0.93–2.63	1.20 [1.14; 1.35]	1.02–1.77	0.1880
SB, c.u.	0.707 [0.666; 0.766]	0.58–1.70	0.676 [0.638; 0.728]	0.58–0.99	0.0705

*—differences between subgroups are statistically significant, *p* < 0.05.

### 2.6. Complex Influence of Saliva Composition Indicators Depending on Sex

**Figure 11:**
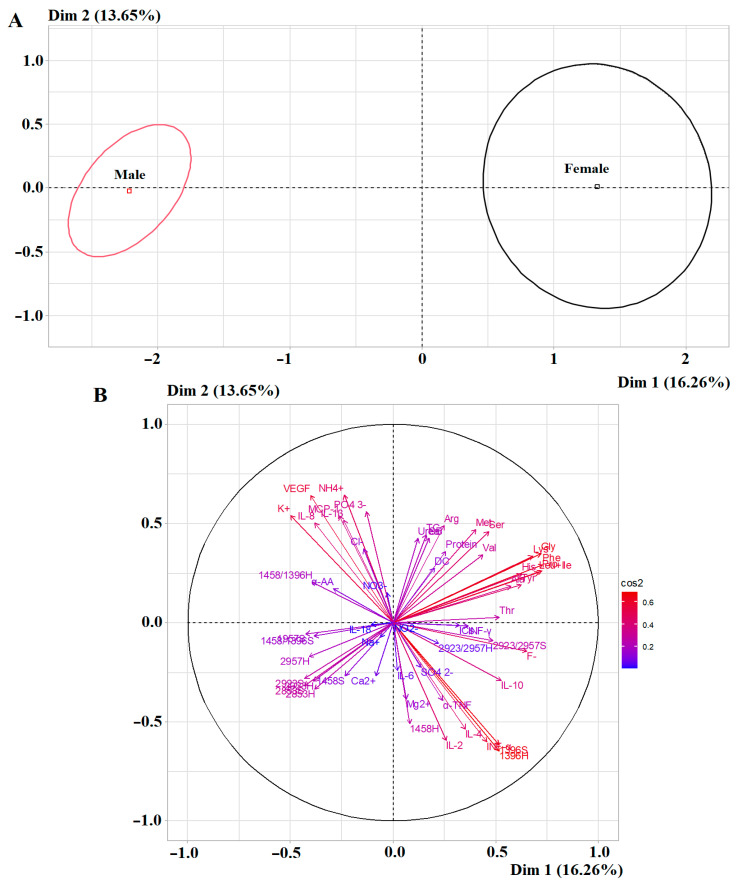
PCA factorial diagram (**A**) and correlation circle (**B**) comparing all salivary indicators depending on sex (*p* = 2.068 × 10^−10^).

During the comprehensive assessment of saliva parameters, a high degree of reliability of separation by the principal component analysis was maintained (Figure 11A). Moreover, high correlation coefficients for the first principal component corresponded to the amino acids Phe (*r* = 0.7301), Pro (*r* = 0.7245), Gly (*r* = 0.7216) and Leu+Ile (*r* = 0.7116) (Figure 11B). For the second principal component, high correlation coefficients were not established; however, medium-strength correlation coefficients were shown for ammonium ions (*r* = 0.6431), VEGF (*r* = 0.6415) and the intensity of the 1396 cm^−1^ absorption band (*r* = −0.6467) (Figure 11B).

## 3. Discussion

Electrolytes play a crucial role in maintaining oral homeostasis and are involved in salivary function, so changes in their concentrations lead to imbalances and pathological conditions [22,23]. The results show that statistically significant differences are most pronounced for electrolyte levels in male and female saliva. Moreover, the concentration distribution in the female group is more variable than in the male group. This can likely be explained by differences in hormonal levels, with the levels more stable in males [24]. In females, electrolyte levels can vary depending on the menstrual cycle, with changes occurring during the follicular and luteal phases [25]. These fluctuations may be due to cyclical fluctuations in plasma estradiol levels [26]. Sodium and potassium levels vary greatly depending on sex, which can be explained by the size of the parotid and submandibular glands, which are larger in males [27]. Furthermore, the rate of saliva flow, which is also associated with the size of the salivary glands, contributes to an increase in the concentration of these ions [28]. When comparing the rate of saliva flow between the sexes, the influence of steroid hormones on salivation in females cannot be ignored. During menopause, with estrogen deficiency, there is a significant decrease in the rate of saliva secretion and an increase in its viscosity [29]. An important factor is the constancy of the Na/K and Ca/P ratios regardless of sex, indicating a balance of processes in the oral cavity.

Magnesium content is more stable in both groups, since these ions enter the saliva from the blood and are not related to the size of the salivary glands. Normally, in adult males and females, the magnesium level in mixed saliva is 0.4–0.9 mmol/L, and in the elderly it may be slightly higher [30]. The body has special molecular mechanisms that maintain the concentration of magnesium in the blood plasma within a certain range (0.7–1.2 mmol/L) by regulating magnesium exchange with cells and reabsorption in the kidneys. Proteins TRPM6 and TRPM7 (transient receptor potential cation channel) [31] and CASR (Ca^2+^/Mg^2+^-sensitive receptor) regulate magnesium reabsorption in the renal tubules. With reduced magnesium levels in the plasma, magnesium reabsorption in the kidneys increases, while with excess magnesium concentration in the blood plasma, reabsorption is significantly reduced. In this way, a certain range of magnesium ion concentrations in the blood plasma is maintained dynamically. It should be noted that magnesium exchange between tissue cells and blood plasma is a slow process (hours to days), while magnesium reabsorption in the kidneys is a much more intense process (minutes to hours). Therefore, due to the existence of special mechanisms regulating magnesium concentration in plasma, magnesium levels in biological fluids and tissues can be significantly depleted against the background of “normal” magnesium levels in the blood plasma. From a physiological point of view, a diagnosis of magnesium deficiency is impossible based solely on the results from measuring magnesium levels in the blood; an assessment of the clinical symptoms of magnesium deficiency and, possibly, additional information (e.g., magnesium levels in daily urine, saliva, and hair) are required. A magnesium distribution coefficient has been proposed, which is the ratio of the magnesium level in the blood serum to the magnesium concentration in saliva (mmol/L); it can be used for the early diagnosis of latent magnesium deficiency [32].

The levels of most amino acids in saliva were relatively high, which is consistent with the literature data [33,34,35]. However, according to the literature data, the levels of various amino acids vary widely, which is due, among other things, to the small size of samples and their heterogeneity. This, in turn, imposes limitations on the criteria for sample formation and calculation of its size to obtain correct and reproducible results. Amino acids can enter the saliva from the blood due to ultrafiltration, passive diffusion and active transport, as well as from the excretory ducts of the salivary glands during the biosynthesis of protein secretion in acinar cells using sodium-dependent membrane transporters. The size and functional activity of the salivary glands affect the volume and composition of saliva secreted into the oral cavity [27]. As the primary secretion passes through the ducts, active transport processes occur which change the composition of saliva. The concentration of amino acids in saliva also changes depending on the rate of salivation and the type of stimulation of salivary flow. A clear reason for the higher amino acid content in saliva in the female subgroup has not been established; further research in this area is required.

Mixed saliva contains specific factors protecting the oral cavity, the most significant of which are immunoglobulins A, M, G, E, secretory immunoglobulin A and lysozyme. They enter the saliva as a result of local synthesis by plasma cells and from the blood by transudation through the gingival groove. The main one in the oral cavity is secretory immunoglobulin A (sIgA), which is synthesized directly by the epithelial cells of the salivary gland ducts [36]. The literature contains various data on the influence of sex on the cytokine content in saliva. There is evidence that sex does not affect the concentration of sIgA, IL-1β, CRP [7,24]. Other sources noted that higher concentrations of IL-6, IL-12, IL-1β and TNF-α and lower levels of IL-2 were found in men compared to women [37,38]. Concentrations of 48 cytokines, chemokines, and growth factors (CCGFs) also differed between male and female groups for some CCGFs in plasma, saliva, and urine [39]. It should be noted that the available literature examples relate mainly to patients; information on healthy individuals is limited [40].

Based on the data we obtained, it is clear that in the male group, a statistically significant increase in concentration was observed only for IL-1β and IL-10. In other cases, changes in concentrations by sex were not found. This may be due to the fact that we examined a group of healthy individuals without any diseases or pathological processes occurring either in the oral cavity or throughout the body. It should be noted that the content of cytokines in saliva does not correlate with their level in the blood. This indicates certain autonomy of local immunity in the oral cavity and reflects the general trends of the cytokine cascade in the patient’s body [41,42,43]. Cytokines can enter saliva from the following sources: lymphocytes and accessory cells of the immune system embedded in the epithelium of the mucous membranes; serum transudate penetrating through the gingival pockets [44]; salivary glands, in which cytokines are formed and enter saliva with their secretions; epithelial cells of the oral mucosa that produce cytokines upon contact with microorganisms [45].

Lipids are among the most important cellular components of human saliva [46,47]. The lipidomic analysis of saliva revealed the presence of mono-, di-, and triglycerides, free fatty acids, squalene, phospholipids, wax esters, and cholesterol esters [48,49]. Saliva contains significant amounts of surface-active phospholipids, in particular phosphatidylcholine (PC), the physiological functions of which in the oral cavity remain to be elucidated [50]. In other parts of the gastrointestinal tract, PC is known to form a protective hydrophobic layer that plays a key role in maintaining the barrier properties of tissues, protecting them from the effects of factors such as acid and microbes.

Lim et al. showed that 385 salivary lipid species were associated with sex [51]. Sphingolipids with the d18:2 base were more strongly associated with sex than other lipid species, especially sphingomyelin SM(d18:2/14:0). Almost all glycerophospholipids esterified with docosahexaenoic acid were positively associated with being female. Previously, it was shown for blood plasma that males are characterized by a more atherogenic lipid profile than females, with a significantly lower level of the HDL fraction [52]. Sales et al. found that sex is the main lipidomic factor, the influence of which is mediated by sex hormone-binding globulin [53]. In epidemiological studies of the lipidome, mixed sex cohorts should be avoided, and women taking hormonal contraceptives should be considered as a separate subgroup.

The lipid composition of saliva is determined by its origin from different salivary glands. Thus, more than half of the lipids secreted by the parotid and submandibular salivary glands are non-polar, while the labial salivary glands secrete a larger amount of lipids, as well as polar lipids such as phospholipids and glycolipids [54,55]. Individual differences in lipolytic activity in the oral cavity may influence the presence and detection of lipids in the oral cavity in different people [56]. Interestingly, analysis of the lipid profile of dental plaque revealed predominantly polar lipids, with approximately 50% of all lipids consisting of triglycerides and phospholipids [57]. These results indicate that dental plaque may be a possible source of polar lipids in the oral cavity. The observed differences in the lipid absorption bands corresponding to vibrations of methyl and methylene groups in the structure of saturated and unsaturated lipids depending on sex may be due to the contribution of different salivary glands to the formation of the secretion, which requires further verification. Previously, differences in male and female spectra were shown using Raman spectroscopy [58]. In particular, male spectra differ from female spectra due to an increased response in the bands of 630, 760 and 1003 cm^−1^, while female spectra demonstrate an increased response at frequencies of 855 and 1300–1400 cm^−1^. Minor differences are also noticeable at shifts of 1051 and 1455 cm^−1^. These changes are associated with the influence of estrogen on the salivary metabolome. We have also previously shown that the infrared spectra of saliva also show differences by sex, which must be taken into account in comparative studies [59].

It is known that biochemical parameters of saliva differ depending on sex; salivary pH, buffer capacity, protein content, MUC5B, secretory IgA and chitinase activity were lower in women than in men, while MUC7 and lysozyme activity were higher [21]. Since saliva is a rapidly changing dynamic environment, it can potentially be used for the long-term monitoring of the body’s condition [8]. Thus, the total protein content reflects the state of protein metabolism in the body, and deviations in the total protein level from the norm can be caused by both physiological and pathological processes. Higher protein concentrations were found in women than in men [60]. Functional studies of saliva have documented dynamic changes in protein composition and protective properties in response to dietary challenges, demonstrating that salivary constituents actively contribute to oral defense and can vary with external stimuli [61]. Xiao et al. identified 82 sex-specific proteins, mainly associated with inflammation, immune function and lipid metabolism, in the composition of saliva [62]. Sun et al. [63] and Flessing et al. [64] found 14 salivary proteins, the expression of which differed in female and male groups. Interestingly, these sex-specific proteins were primarily involved in acute phase signaling and LXR/RXR activation. For example, a sex-specific protein (A2M) was identified, which is an acute phase protein involved in the complement pathway and the blood coagulation cascade, which is also associated with immune function [65].

This means that different biological fluids may, to some extent, exhibit similar proteomic sex differences. Differences in the proteomic profiles of male and female saliva may result in differences in integral parameters, such as the total content of α-amino acids and imidazole compounds, free amino acids, and urea as end products of protein metabolism. It cannot be ruled out that the identified differences may be due to differences in the composition of the oral microbiome, as sex differences have been established in the salivary microbiota of healthy individuals [66]. Zaura et al. assessed whether the salivary microbiome and metabolome could be separated by sex. Sixty-five taxonomic units were identified that differed between men and women, of which 44 taxonomic units (including 19 streptococcal) were significantly more common in female saliva, while *Veillonella*, *Prevotella*, and *Megasphaera* predominated in the microbiome of male saliva [67]. There were no differences in the Shannon diversity index between volunteers of different sex.

There are sex differences in lipid hydroperoxide levels, with lipid peroxidation processes being more pronounced in females [68]. These differences are associated with different mechanisms of antioxidant defense: females have a more powerful antioxidant system, while males have a less powerful one. Male sex hormones (androgens) have virtually no antioxidant activity, unlike estrogens [69]. However, differences in lipid peroxidation product levels in healthy controls are not statistically significant, consistent with published data.

Limitations of the study include the limited age range (middle-aged adults), while the generalizability of the results to younger and older populations remains to be determined. The oral microbiota was not assessed, despite its known role in amino acid metabolism and the regulation of salivary acidity. This is planned for follow-up research. The sample size and diversity require attention, as larger and more diverse groups could contribute to the creation of global reference ranges.

## 4. Materials and Methods

### 4.1. Study Design

The study involved healthy volunteers (75 women, average age 40.6 [33.7; 45.7] years; 45 men, average age 45.4 [39.5; 50.9] years). Differences in age between subgroups were statistically insignificant (*p* > 0.05). The group was recruited from the blood transfusion department of the Omsk Clinical Oncology Dispensary. The inclusion criterion was the absence of clinically significant somatic pathologies, including diabetes mellitus, cardiovascular and oncological diseases, etc. All study participants had a confirmed absence of active caries and inflammatory processes in the oral cavity.

### 4.2. Methods of Collection, Storage, Transportation and Pre-Processing of Saliva

Saliva samples were collected after an overnight fast and preliminary mouth rinse without additional stimulation in sterile polypropylene centrifuge tubes (saliva volume 5–6 mL). Saliva was collected between 8 and 10 am at one time of year (October–November) without dietary control. Salivation rate (mL/min) was calculated in all cases. Due to the lack of differences in salivation rate between subgroups, no further normalization for this parameter was performed. Samples of unsatisfactory quality were rejected at the collection stage. From collection until delivery to the laboratory, samples were stored at 2–8 °C for no more than 4 h. The samples were then centrifuged at 10,000× *g* (CLb-16, Moscow, Russia) for 10 min to separate the sediment and reduce turbidity. The supernatant saliva was transferred to four Eppendorf tubes and frozen at −86 °C until analysis.

All saliva samples were analyzed for biochemical parameters (75/45), cytokines (44/45), lipids (59/45), amino acids (71/45), cations, and anions (54/45 for the female and male subgroups, respectively). Differences in sample size in the female subgroup were due to insufficient saliva volume in some cases.

### 4.3. Method for Determining Cations and Anions in Saliva

The content of cations and anions in saliva was determined by capillary electrophoresis (KAPEL-105M, Lumex, St. Petersburg, Russia). The measurement method is based on filtration, dilution of the collected sample, subsequent separation, and quantitative determination of components with indirect detection at a specific wavelength. We selected the following conditions for determining the cationic and anionic composition of saliva: a 100 µL aliquot of the test sample, diluted 20-fold with bidistilled water.

To determine cations (ammonium, potassium, sodium, magnesium, and calcium ions), the leading electrolytes were 20 mM benzimidazole, 5 mM tartaric acid, and 2 mM 18-crown-6. To determine anions (chlorides, nitrites, nitrates, sulfates, fluorides, and phosphates), the leading electrolytes were 10 mM CrO_3_, 30 mM diethanolamine, and 2 mM cetyltrimethylammonium hydroxide. Fluka reagents (Buchs, Switzerland) were used.

A quartz capillary with L_ef_/L_total_ = 50/60 cm and internal diameter (ID) 75 µm was used. Immediately before the analysis, the capillary was washed with distilled water for 3 min, 0.5 M sodium hydroxide solution for 5 min, distilled water for 5 min, and a leading electrolyte solution for 10 min. The sample was introduced into the capillary pneumatically (30 mbar, 10 s). The constant voltage was 25 kV for determining cations and −17 kV for anions. The wavelength of the photometric detector was 267 nm for cations and 374 nm for anions. The experiment was carried out at 20 °C. The analysis time in both cases was 6–7 min.

### 4.4. Method for Determining the Amino Acid Composition of Saliva

The analysis was performed using a KAPEL-105M capillary electrophoresis system with a positive polarity high-voltage source (Lumex, St. Petersburg, Russia). The leading electrolyte was a phosphate-buffered solution pH = 7.7–7.8 (phosphates—30 mmol/L; β-cyclodextrin—4 mmol/L). For the study, a quartz capillary L_ef_/L_total_ = 65/75 cm, ID = 50 μm was used. Sample introduction into the capillary was pneumatic (30 mbar, 5 s), with constant voltage—25 kV, temperature +30 °C, and separation time 17–18 min, and the working wavelength of the photometric detector was 254 nm.

To analyze free amino acid content, the phenylisothiocarbamyl derivatives were first prepared. A 300 μL saliva sample was mixed with 150 μL of sodium carbonate solution (0.1 mol/L) and 300 μL of phenyl isothiocyanate (FITC) in isopropanol. The mixture was thoroughly mixed until the precipitate dissolved, left at room temperature for 35 min, and then evaporated to dryness under a stream of warm air. The 35-min exposure time ensures complete interaction of FITC with the N-terminal amino acid of the peptide without excessive modification of other regions of the molecule. This allows for the formation of stable FITC derivatives that can be isolated and identified without degradation. Isopropanol ensures sufficient solubility of FITC and its uniform distribution in the reaction mixture during this time. Dry residues were dissolved in 500 µL of distilled water and used for analysis during the working day.

Quantitative determination of amino acids was performed using calibration graphs constructed using a ready-made standard solution of amino acid mixture (Merck KGaA, Darmstadt, Germany) (arginine—Arg, lysine—Lys, tyrosine—Tyr, phenylalanine—Phe, histidine—His, leucine—Leu, isoleucine—Ile, methionine—Met, valine—Val, proline—Pro, threonine—Thr, serine—Ser, alanine—Ala, glycine—Gly). The accuracy and reproducibility of the results of amino acid determination in saliva samples were confirmed by the “added-found” method. Standard samples of amino acids with known concentrations (at least 3 different concentrations) were added to the same original saliva sample, thus verifying both the correctness of the peak identification on the electropherogram and the accuracy of the concentration calculation. The error in amino acid determination in all cases did not exceed 20%.

### 4.5. Determination of the Lipid Profile of Saliva by Infrared (IR) Spectroscopy

To determine salivary lipids, preliminary extraction of lipids was performed with Folch solution (chloroform: ethanol = 2:1, vol.) according to the method adapted by the authors, followed by analysis of the extracts by infrared (IR) spectroscopy [70]. The intensity (H) of the absorption bands at 1396 cm^−1^ (δCH_3_), 1458 cm^−1^ (δCH_2_), 2853 cm^−1^ (ν_s_CH_3_), 2923 cm^−1^ (ν_as_CH_2_) and 2957 cm^−1^ (ν_as_CH_3_) was analyzed, as well as the ratios 2923/2957 and 1458/1396 cm^−1^ (ν_s_—symmetric stretching, ν_as_—asymmetric stretching and δ—deformation vibrations) [71].

### 4.6. Determination of Salivary Cytokine Concentrations

The content of salivary cytokines IL-1β (cat. No. A-8766), IL-2 (cat. No. A-8772), IL-4 (cat. No. A-8754), IL-6 (cat. No. A-8768), IL-8 (cat. No. A-8762), IL-10 (cat. No. A-8774), IL-18 (cat. No. A-8770), INF-α (cat. No. A-8758), INF-γ (cat. No. A-8752), MCP-1 (cat. No. A-8782), and TNF-α (cat. No. A-8756) and vascular endothelial growth factor (VEGF, cat. No. A-8784) was determined by solid-phase enzyme-linked immunosorbent assay (Vector Best, Novosibirsk, Russia) on a Thermo Fisher Multiskan FC analyzer (Waltham, MA, USA). The aliquot volume in all cases was 100 μL; the units of measurement for MCP-1, TNF-α, IL-1β, IL-2, IL-4, IL-6, IL-8, IL-10, IL-18, INF-α, and INF-γ were pg/mL, and for VEGF mU/mL. All analyses were performed according to the manufacturer’s instructions, without modification to reagent or sample volumes. Concentrations were calculated in all cases using a pre-generated calibration curve.

### 4.7. Determination of the Biochemical Composition of Saliva

In all saliva samples, the following were determined: total protein by reaction with pyrogallol red (mg/L, cat. No. B-8084, Vector-Best LLC, Novosibirsk, Russia), urea by the urease-salicylate method according to Berthelot (mmol/L, cat. No. B-8074, Vector-Best LLC, Novosibirsk, Russia), total content of α-amino acids by reaction with ninhydrin (α-AAs, mmol/L), and imidazole compounds by the diazotization reaction in the presence of sulfanilic acid (ICs, mmol/L) using the StatFax 3300 semi-automatic biochemical analyzer (Awareness Technology, Palm City, FL, USA) [72]. The content of lipid peroxidation products (diene conjugates—DC, triene conjugates—TC, Schiff bases—SB, c.u.) was determined using the Volchegorsky method [73].

The validation procedure for each test system included two analytical runs. Each run included the analysis of calibration standards to construct a calibration curve, as well as the required number of quality control samples with a specified concentration of the corresponding indicator. Each sample was analyzed in duplicate.

### 4.8. Statistical Analysis

The results were processed nonparametrically using Statistica 13.3 EN software (StatSoft, Tulsa, OK, USA). The results are presented as the median and interquartile range. Principal component analysis (PCA) was performed using the PCA program in R (RStudio, version 3.2.3, Boston, MA, USA). The PCA results are presented as factor planes and corresponding correlation circles. In each case, only the first two principal components (Dim 1 and Dim 2) are shown in the figures. The color of the arrows on the correlation circle changed from blue (weak correlation) to red (strong correlation), as shown in the color bar. The orientation of the arrows characterizes positive and negative correlations (for the first principal component, we analyzed the arrows’ position relative to the vertical axis; for the second principal component, we analyzed the arrows’ position relative to the horizontal axis).

## 5. Conclusions

A pairwise comparison of the composition of mixed saliva revealed that sex differences were most pronounced in amino acid content and lipid absorption bands in the IR spectra of saliva. A number of individual differences were observed for electrolyte and biochemical compositions; however, the ranges of variation for all indicators overlapped, consistent with the general normal range. Although overall cytokine profiles did not show clear multivariate separation, significant differences between sexes were observed for individual cytokines. A principal component analysis demonstrated that a comprehensive assessment of all parameters in each group allows for differentiation between male and female subgroups, with the exception of cytokines. Combining all salivary parameters allows for the creation of a unique sex-associated metabolic profile of saliva. Despite the statistical separability of subgroups, the biological or clinical significance of the differences requires further clarification. Overall, understanding the differences in normal saliva composition will allow for the appropriate formation of patient cohorts for various research purposes. In particular, mixed cohorts should be avoided when analyzing salivary amino acid and lipid profiles.

## Figures and Tables

**Table 1:** Content of cations and anions in saliva.

Indicator	Female, *n* = 54		Male, *n* = 45		*p*-Value
	Me [IQR]	Min-Max	Me [IQR]	Min-Max	
NH_4_^+^, mg/L	105.4 [70.56; 170]	17.4–257.1	168.9 [129.3; 207.3]	44.4–363.4	0.0001 *
K^+^, mg/L	485.8 [395.7; 603.6]	241.1–877.8	636.5 [609.2; 706.0]	465.9–1208.0	<0.0001 *
Na^+^, mg/L	128.3 [92.53; 163.3]	44.6–920.9	171.6 [133.4; 247.0]	78.5–1349.0	0.0009 *
Mg^2+^, mg/L	3.51 [2.41; 4.30]	1.10–12.24	2.25 [1.91; 3.78]	0.95–8.75	0.0060 *
Ca^2+^, mg/L	46.40 [30.25; 60.99]	14.06–107.30	40.28 [26.71; 59.94]	13.07–99.15	0.3287
Cl^−^, mg/L	546.6 [438.6; 694.6]	219.3–1254.0	537.4 [464.9; 617.2]	383.6–1535.0	0.8166
NO_2_^−^, mg/L	2.07 [0.43; 3.57]	0.00–17.69	1.01 [0.00; 3.47]	0.00–28.23	0.0983
SO_4_^2−^, mg/L	27.51 [21.48; 34.06]	9.16–647.00	16.54 [13.09; 21.53]	6.30–51.14	<0.0001 *
NO_3_^−^, mg/L	2.38 [1.45; 4.91]	0.00–29.15	1.49 [0.76; 4.34]	0.00–40.07	0.1239
F^−^, μg/L	3.06 [1.44; 5.14]	0.51–22.63	0.99 [0.73; 1.35]	0.14–3.76	<0.0001 *
PO_4_^3−^, mg/L	273.0 [225.3; 342.4]	141.8–593.1	305.9 [279.6; 383.7]	113.2–562.7	0.1179
Ca/P, mg/L	0.353 [0.246; 0.518]	0.122–1.133	0.294 [0.190; 0.503]	0.105–1.047	0.1662
Na/K, mg/L	0.422 [0.316; 0.662]	0.122–3.619	0.394 [0.313; 0.654]	0.191–3.141	0.8882

Note. Abbreviations (here and in Table 2, Table 3, Table 4 and Table 5): Me—median; IQR—interquartile range (25–75%); min—minimum value; max—maximum value; *—differences between subgroups by sex are statistically significant, *p* < 0.05.

## Data Availability

The raw data supporting the conclusions of this article will be made available by the authors on request.
